# Development of Pulsed Eddy Current Nondestructive Testing: A Review

**DOI:** 10.3390/s26082289

**Published:** 2026-04-08

**Authors:** Qian Huang, Ruilin Wang, Jingxi Hu, Hao Jiao, Chi Zhang, Zhitao Hou, Chenxi Duan, Xueyuan Long, Liangchen Lv

**Affiliations:** 1 School of Petroleum Engineering, Chongqing University of Science and Technology, Chongqing 401331, China; 2 School of Safety Science and Engineering, Chongqing University of Science and Technology, Chongqing 401331, China; 3 Shunan Gas Mine, Southwest Oil and Gas Field Branch of PetroChina, Luzhou 646000, China

**Keywords:** nondestructive testing, PECT sensor, pulsed eddy current testing, sensor, signal processing

## Abstract

As a branch of nondestructive testing (NDT), Pulsed Eddy Current Testing (PECT) is characterized by its wide frequency spectrum and high penetration depth. After years of development, it has been widely applied to defect detection and material characterization of key components in industries such as petrochemicals, new energy, and aerospace. With the large-scale application of new energy sources like liquefied natural gas (LNG), methanol, and liquid hydrogen, the demand for NDT of non-ferromagnetic materials (e.g., austenitic stainless steel) has surged. However, challenges such as electromagnetic leakage caused by low magnetic permeability and the lift-off effect induced by protective layers impose stricter requirements on inspection technologies, driving the evolution of PECT towards adaptability in complex scenarios. This paper systematically reviews the latest advances in PECT technology, covering detection sensors, modeling methods, detection signal processing, and engineering applications. With a particular emphasis on research outcomes from the past decade, this paper also proposes potential directions for future development, aiming to provide a reference for innovative research and the industrial promotion of PECT technology.

## 1. Introduction

Pulsed Eddy Current Testing (PECT) has evolved over more than half a century since it was first proposed by Waidelich [1] in the 1950s for measuring the thickness of metal coatings on nuclear fuel elements. Subsequently, the technology has seen further development and application in the aerospace, petrochemical, and power industries. In 1997, the Dutch company RTD developed the RTD-INCO TEST equipment [2], designed for detecting corrosion in ferromagnetic materials with insulation layers, capable of inspecting wall thicknesses ranging from 3 to 65 mm. In 2019, Eddyfi (Canada) introduced the Lyft instrument [3], which increased the detectable wall thickness up to 38 mm. Compared to traditional Eddy Current Testing (ECT), PECT offers unique advantages, particularly in the inspection of new industrial materials, and has attracted the attention of researchers worldwide.

**Figure 1:**
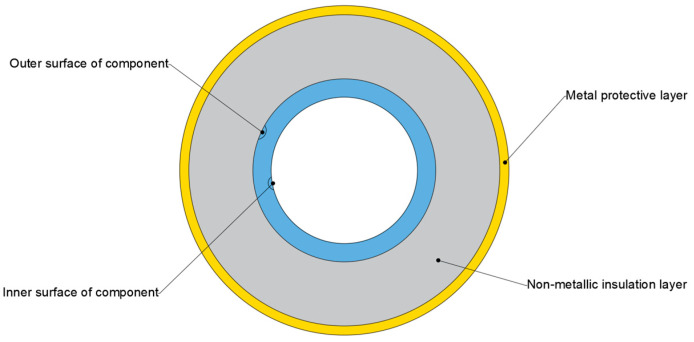
Schematic diagram of corrosion in a pipeline with a protective layer.

In industrial sectors, particularly the petrochemical industry, protective layers are typically applied to component surfaces (such as pipelines) to prevent direct contact with the external environment and avoid economic losses, as shown in Figure 1. Depending on application requirements, these protective layers can be categorized into non-metallic insulation layers for thermal insulation and metallic cladding for corrosion protection. During the service life of these components, thickness loss due to corrosion is a primary cause of failure. Unlike Ultrasonic Testing [4], Radiographic Testing [5], and Infrared Thermography [6], PECT does not require the removal of external protective layers or the shutdown of the equipment under test. It enables inspection under non-contact conditions and remains unaffected by surface conditions. However, its penetration capability is limited by the material and thickness of the protective layer; for example, a thick ferromagnetic protective layer can cause severe attenuation of the detection signal, thereby restricting the effective inspection of the component.

With the widespread adoption of new energy sources such as liquid hydrogen and liquefied natural gas (LNG), storage and transportation materials are shifting from traditional ferromagnetic metals (like carbon steel) to non-ferromagnetic metals (such as austenitic stainless steel). These materials possess excellent corrosion resistance and are commonly used for cryogenic pipelines. Given the stringent storage requirements and complex equipment associated with these energy sources, leakages can lead to severe hazards including frostbite, asphyxiation, fire, and explosion. Consequently, the application of PECT to non-ferromagnetic metal pipelines holds greater practical significance than for ferromagnetic pipelines, creating an urgent demand for further research in this area.

The core advantage of PECT lies in its broader frequency spectrum, which contains a richer set of frequency components. Compared to traditional sinusoidal excitation, the use of square wave or step excitation results in a wider spectrum. The low-frequency components within this spectrum can achieve greater penetration depth, thereby overcoming the limitations imposed by the skin effect in traditional eddy current testing. Given the frequency dependence of the skin effect, virtually all induction-based eddy current detection methods benefit from these broadband characteristics.

**Figure 2:**
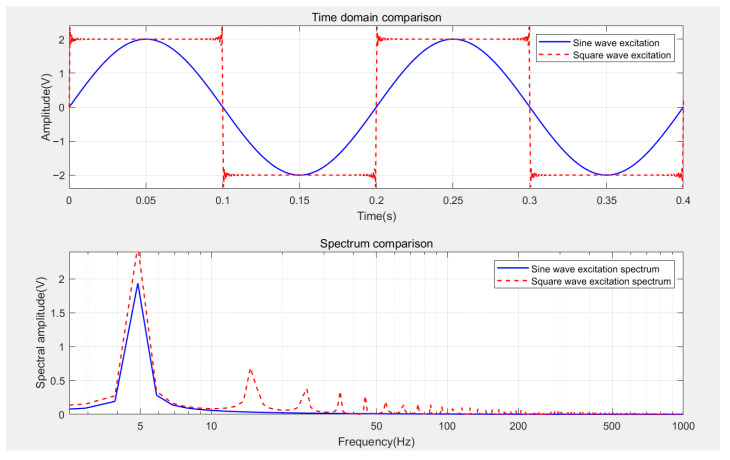
Comparison of different excitation signals in the time and frequency domains.

Traditional ECT utilizes sinusoidal excitation with a narrow spectrum and a fixed skin depth. To detect defects at varying depths, it often requires the sequential application of different excitation frequencies. In contrast, the square wave excitation used in PECT allows for the simultaneous detection of defects at multiple depths in a single inspection. Unlike the rapid attenuation of sinusoidal waves in the frequency domain, square wave excitation maintains significant energy intensity across multiple harmonic frequencies. Figure 2 illustrates the differences between sinusoidal and square wave excitations in both the time and frequency domains.

Compared to traditional eddy current sensors, PECT sensors generate a stronger magnetic field with wider coverage. This capability effectively mitigates the impact of the lift-off effect while enabling the detection of metal corrosion over larger areas.

Driven by emerging demands such as the new energy sector, PECT technology has experienced rapid development over the past decade. Therefore, the primary objective of this paper is to introduce the latest advancements in PECT technology, thereby promoting its application in resolving modern engineering bottlenecks (e.g., the inspection of non-ferromagnetic storage and transportation facilities). Concurrently, this paper systematically summarizes and classifies the technological breakthroughs achieved over the past ten years. By combining the popularization of recent progress with a structured taxonomy, this review aims to provide researchers and field engineers with a practical guide to the comprehensive landscape of state-of-the-art PECT technology.

The remainder of this paper is organized as follows: Section 2 briefly describes the basic principles of PECT. Subsequent sections provide a systematic review of sensors, modeling methods, and signal processing techniques. Finally, the paper concludes with a summary of the technology’s status and future outlook.

## 2. Principle of Pulsed Eddy Current Testing

**Figure 3:**
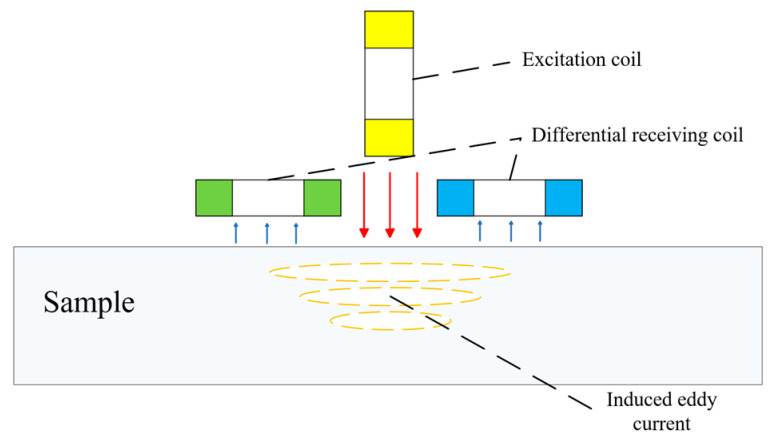
Schematic diagram of PECT using a differential probe.

In PECT, the excitation coil is energized with a square wave or pulsed current. Based on the principle of electromagnetic induction, this creates a primary magnetic field that induces eddy currents in the conductive object under test, as illustrated in Figure 3. These eddy currents generate a secondary magnetic field carrying information about the object’s properties, such as wall thickness. When defects are present, both the induced eddy currents and the resulting magnetic field undergo corresponding changes. By designing appropriate receiving (pickup) elements to detect these signals and employing suitable methods, the condition of the object can be assessed.

A critical factor in PECT is the penetration depth of the induced eddy currents within the object under test, as this dictates the effective inspection range. The penetration depth is governed by the skin effect, which describes the depth at which the current density attenuates to 1/e of its surface value. Typically denoted by δ, this parameter characterizes the rate of electromagnetic field decay and is mathematically expressed as:

(1) $$\delta =\sqrt{\frac{2}{\omega \mu \sigma }}$$

where *σ* represents the skin depth (m), *ω* is the angular frequency (rad/s), *μ* is the magnetic permeability (H/m), and *σ* is the electrical conductivity (S/m). As indicated by Equation (1), for a material with constant conductivity and permeability, the skin depth depends solely on the excitation frequency. Consequently, by varying the excitation frequency or utilizing an excitation signal containing multiple frequency components, the limitations imposed by the skin effect can be mitigated, allowing for the acquisition of more comprehensive information about the component.

**Figure 4:**
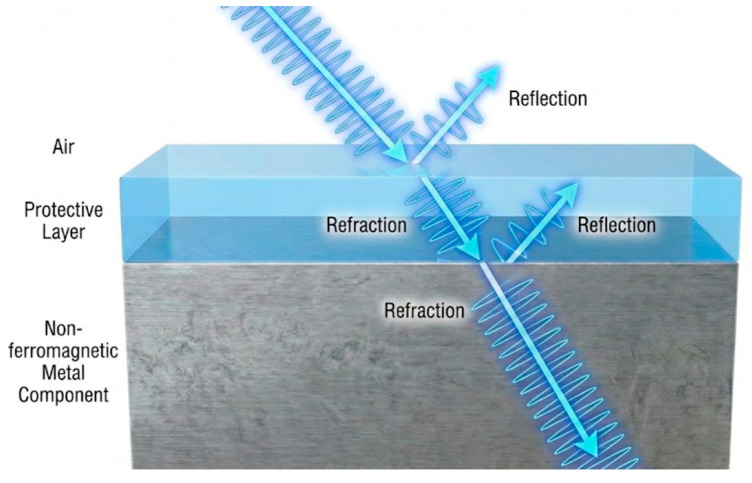
Deflection of electromagnetic waves during propagation.

In contrast to ferromagnetic materials, non-ferromagnetic materials possess lower magnetic permeability and exhibit poor magnetic field focusing capabilities. During inspection, electromagnetic waves generated by the excitation undergo reflection and refraction at the interface between the medium and the pipeline (typically an air-pipeline interface), as illustrated in Figure 4. The angle of refraction is determined by the relative permeability of the object under test. Although this phenomenon parallels light propagation in optical inspection, PECT aims to minimize electromagnetic refraction rather than exploit it.

For ferromagnetic materials, the relative permeability is significantly greater than 1, causing the refraction angle to approach zero. Consequently, magnetic field lines incident at any angle are refracted to become perpendicular to the surface, demonstrating the magnetic focusing effect characteristic of ferromagnetic materials. Conversely, for non-ferromagnetic materials, the relative permeability is approximately 1, meaning the magnetic field direction remains largely unchanged. This results in higher electromagnetic leakage during inspection. This issue is further complicated by the presence of various protective layers commonly found in industrial applications. The additional interfaces introduced by protective layers, combined with the effects of pipeline curvature and lift-off distance, lead to severe attenuation of electromagnetic energy. This makes received signal processing challenging and imposes stricter performance requirements on PECT systems.

**Figure 5:**
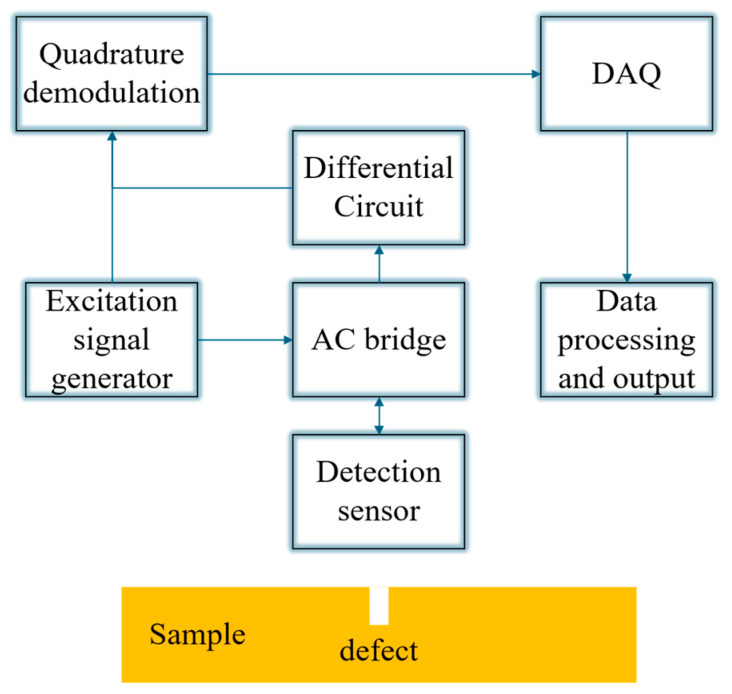
Block diagram of a PECT system using a differential probe.

Various PECT system configurations have been developed to meet diverse application requirements. Figure 5 presents the block diagram of a PECT system utilizing a differential probe. The system is driven by an excitation signal generator, which supplies the primary signal to the alternating current (AC) bridge (integrated with the detection sensor) and a reference signal to the quadrature demodulation module. When the probe passes over a defect, the weak anomaly signal generated by the bridge unbalance is extracted by the differential circuit, thereby effectively suppressing common-mode noise. Subsequently, the signal is processed by the quadrature demodulation module to extract key amplitude and phase features, before finally being output by the data acquisition (DAQ) system for analysis.

The distinctions among different PECT systems are primarily manifested in the design of the detection sensors and the methods used for signal processing. These can be further categorized into four key aspects: the excitation coil, the receiving element, signal preprocessing, and feature extraction.

## 3. Detection Sensors

**Figure 6:**
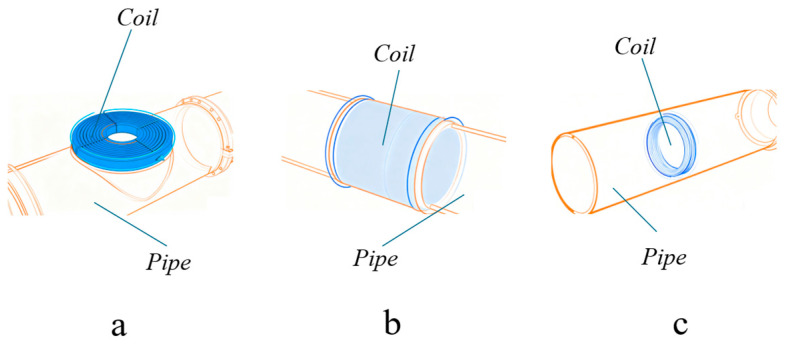
Schematic diagrams of three types of excitation coils: (**a**) surface coil; (**b**) encircling coil; (**c**) internal coil.

PECT sensors generally consist of an excitation coil and a receiving element. The excitation coil is designed to generate induced eddy currents within the object under test, while the receiving element captures the electromagnetic response returned from the object; these components can function as separate entities or be integrated into a single unit. Based on their physical arrangement relative to the test object, excitation coils can be classified into surface coils (also known as pancake coils), encircling coils (or outer diameter coils), and internal coils (or inner diameter coils) [7], as shown in Figure 6. Surface coils are suitable for inspecting planar or curved surfaces, such as the exterior walls of pipelines or steel plates; encircling coils are applied to the inspection of cylindrical structures like pipes; and internal coils are designed for inspecting the interior surfaces of hollow cylindrical structures. Compared to conventional circular coils, rectangular excitation coils can induce a more uniform eddy current distribution within the object, thereby offering higher sensitivity to defects. Zhang et al. [8,9] utilized rectangular coils to characterize the depth and size of defects, confirming that such coils produce more uniform eddy currents in the specimen.

Compared to excitation coils, there is a wider variety of options for receiving elements. High-sensitivity magnetic sensors are commonly selected, such as induction coils, Hall sensors, Superconducting Quantum Interference Devices (SQUIDs) [10], and Magnetoresistive (MR) sensors. MR sensors can be further classified into Giant Magnetoresistive (GMR) [11], Tunnel Magnetoresistive (TMR) [12], and Anisotropic Magnetoresistive (AMR) [13] devices.

SQUIDs offer the highest sensitivity, with a detection limit as low as 10–15 T. Their detection depth and size range surpass those of other element types, enabling the capture of even weak eddy current responses in non-ferromagnetic materials. However, the application of SQUIDs in PECT is limited by their high cost, the complexity of supporting equipment, and poor immunity to interference caused by their high sensitivity.

Although AMR sensors have lower sensitivity and are generally capable of capturing only larger defects, they remain useful in PECT due to their low cost and strong anti-interference capabilities. Tamhane et al. [14] developed a low-cost PECT system based on AMR sensors, capable of detecting corrosion under low current intensity and large lift-off conditions. Tsukada et al. [15] developed a corrosion detection system for buried steel structures using an AMR probe, which successfully evaluated steel corrosion under excitation frequencies below 100 Hz.

Among various magnetoresistive devices, TMR sensors exhibit the highest sensitivity, fast response times, and good stability—particularly thermal stability, which is superior to other MR sensors. Despite their high cost, TMR sensors have seen certain applications. Meng et al. [16] designed a PECT system based on TMR sensors and employed deep learning methods to achieve high-precision thickness identification, with prediction results remaining unaffected by interference factors such as lift-off and edge effects. Mohamed et al. [17] proposed a novel probe design based on solid-state TMR, using air gaps and thin aluminum layers to simulate insulation and cladding. The system achieved a mean absolute error of 0.19 mm, outperforming existing PECT probes utilizing Hall elements.

In PECT system applications, induction coils, Hall elements, and GMR sensors feature mature manufacturing processes and wide measurement ranges, making them widely adopted. Both Hall elements and GMR sensors measure magnetic field intensity and exhibit higher sensitivity to low-frequency signals. Hall elements possess the widest dynamic range (approximately 100 μT to 100 mT) but suffer from high noise and low resolution. However, the use of microchip-type Hall sensors under low-frequency operating conditions (20 Hz to 10 kHz) significantly reduces intrinsic noise. Furthermore, because their sensitivity exhibits low frequency dependence, they successfully overcome the inherent inefficiency of traditional induction coils at low frequencies [18]. Conversely, GMR sensors are characterized by low noise, high operating frequencies, and sensitivity to weak signals, offering advantages in the detection of minute defects.

Induction coils measure the rate of change of magnetic flux density and demonstrate higher sensitivity to high-frequency signals. Due to their advantages in cost, manufacturing simplicity, and large magnetic field measurement dynamic range, they occupy a dominant position in engineering applications [19].

Regarding sensor optimization, array configurations enable the acquisition of differential signals within a single excitation cycle while simultaneously facilitating rapid scanning of large areas. Common array distribution methods include the tri-axial array, which can simultaneously acquire magnetic field data in three directions [20] but entails a relatively complex structure and higher cost. The linear array, where multiple probes are arranged along a straight line, offers a simple structure and flexible application [21]. Additionally, circumferential arrays [22] and matrix arrays [23] are also utilized.

With the further development of technology and related applications, sensor distribution methods have been continuously optimized. Xie et al. [24] designed a magnetic-force-transmission eddy current array split probe. In this design, the excitation device and receiving sensor are placed inside and outside the pipeline, respectively. The probe components move synchronously under magnetic force, ensuring that detection signals for deeply buried defects do not become saturated. Yu et al. [25] conducted a comparative study on the detection performance of cylindrical and U-shaped probes using both simulation and experimental methods. They found that the U-shaped probe more effectively focuses eddy currents beneath the probe, resulting in higher detection sensitivity.

**Figure 7:**
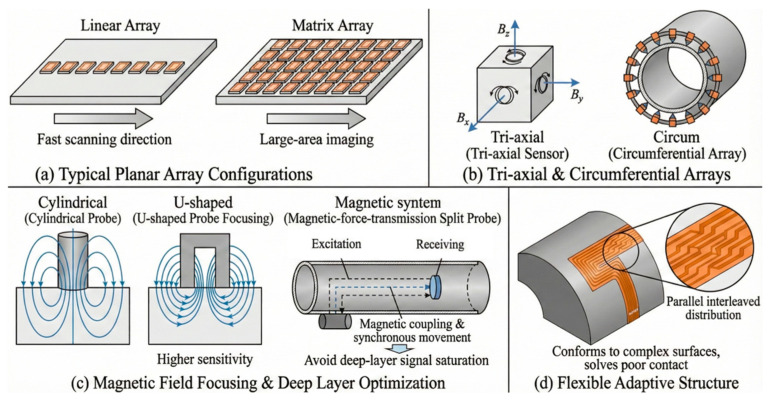
PECT probe structures and array configurations.

Beyond traditional sensor designs, novel sensor structures are continuously emerging. Xie et al. [26] selected induction coils with a wide frequency response range to propose a novel mutual inductance PECT sensor structure. By increasing the proportion of low-frequency indirect coupling energy in the signal, this design achieves high-precision detection of deep subsurface defects. Zhang et al. [27] designed a flexible eddy current array sensor featuring a parallel staggered distribution soldered onto a Flexible Printed Circuit Board (FPCB). This sensor is capable of conforming to complex curved geometries, thereby resolving issues related to poor contact and signal distortion commonly associated with traditional rigid or pen-style probes. Figure 7 shows the structures of several types of PECT sensors.

Regarding sensor excitation, methods primarily utilize either constant current or constant voltage excitation. Constant current excitation is generally superior in performance due to the direct relationship between current and magnetic field, whereas constant voltage excitation is easier to implement in practical applications. Since different excitation modes influence feature extraction in PECT, the appropriate method is typically selected based on specific requirements. As technology advances, simple constant excitation is often insufficient. Researchers now adjust parameters such as the duty cycle, pulse duration, and pulse amplitude to meet requirements for higher precision. Xu et al. [28] combined experimentation with simulation to modify a coil into multiple sub-coils driven by sequential pulses with varying delays. They found that sequential excitation could regulate the diffusion and decay processes of eddy currents within the test object, simultaneously achieving probe miniaturization and signal enhancement. He et al. [29] proposed a coil structure parameter optimization method based on a Kriging surrogate model. Through modeling, sampling calculation, and algorithmic iteration, they improved the response value of the detection signal by 67.2% compared to single-factor optimization methods, significantly enhancing detection sensitivity.

Ferromagnetic materials possess magnetic focusing properties, allowing energy loss to be minimized by adjusting the sensor coverage area. In contrast, non-ferromagnetic materials require larger sensor coverage, making energy loss a more prominent issue [30]. A common approach to address this is optimizing sensor dimensions to improve PECT performance [31,32,33,34]. However, methods based solely on experiments and simulations are often time-consuming and limited to specific object types. Consequently, researchers have explored alternative methodologies. The Response Surface Methodology (RSM) constructs an optimization objective function by fitting a response surface to the variables, significantly reducing workload. Gong et al. [35] applied orthogonal methods, RSM, and the Finite Element Method (FEM) to optimize the shape and parameters of a cone-shaped sensor, finding that the combination of RSM and FEM yielded the most significant results. However, constructed functions may deviate in describing correlations between variables; analytical models, which establish precise coupling relationships between variables, offer higher efficiency and accuracy and are considered ideal for sensor optimization [36]. Song et al. [37] focused on non-ferromagnetic pipelines and approached the problem using an analytical model. They proposed an optimization method for the excitation coil based on the primary magnetic field generated by the excitation current. By decoupling the excitation process from the PECT analytical solution, the model isolated the electromagnetic coupling relationship between the excitation coil and the test object, excluding interference factors and successfully enhancing the proportion of the effective signal. Song et al. also investigated a racetrack-shaped coil suitable for small-diameter non-ferromagnetic pipes, which guided the magnetic field to focus on the pipeline region, thereby improving defect resolution [38].

In engineering applications, non-ferromagnetic materials are often wrapped in protective layers for thermal insulation or corrosion prevention. Compared to bare materials, components with protective layers (such as pipelines) suffer more severely from the lift-off effect. Addressing the gap between pipelines, Klein et al. [39] proposed a sensor structure where the excitation and receiving coils are not coaxial. By adjusting the distance between the center axes of the two coils, they maximized the phase difference between the defect response and the lift-off response, thereby reducing the influence of the lift-off effect.

Despite the aforementioned advancements in sensor design, current PECT technologies still face numerous limitations, particularly when applied to non-ferromagnetic materials in complex working environments. Although high-sensitivity sensors like SQUIDs can capture extremely weak eddy current responses, their practical application is severely restricted by high costs and operational complexity. Furthermore, while structural optimizations—such as analytical model-based coil designs and non-coaxial configurations—can partially mitigate energy loss and lift-off effects, these approaches typically rely on idealized models or specific geometric constraints. In emerging sectors like the new energy industry (e.g., liquefied natural gas pipelines), non-ferromagnetic components are often encapsulated in thick, multi-layered, and complex protective coatings. Under these extreme conditions, the inherently weak secondary magnetic field of non-ferromagnetic materials causes the signal-to-noise ratio of existing sensors to drop to unacceptable levels. Consequently, the inherent limitations of current sensor structures underscore an urgent demand for entirely novel designs.

## 4. Modeling Methods

As a branch of ECT, PECT shares similar modeling methodologies, which are generally categorized into circuit models and electromagnetic field models. Circuit models represent the PECT system as a coupled circuit comprising resistors, inductors, and capacitors, thereby describing electromagnetic relationships indirectly. These models are characterized by simple construction and high computational efficiency, eliminating the need to solve complex equation systems. While suitable for preliminary PECT research, they neglect the spatial distribution of the electromagnetic field. Consequently, they fail to address variations in eddy current depth caused by the skin effect and perform poorly when inspecting complex objects. Electromagnetic field models are based on Maxwell’s equations and solve electromagnetic field equations through specific boundary conditions. They can accurately characterize field distribution laws and effectively reflect process variations. Although widely adopted in research, they remain limited by the complexity of the process and significant computational time costs.

### 4.1. Circuit Models

Circuit models offer simplicity and rapid calculation. In 1996, Loos et al. [40] simplified the electromagnetic field model into a circuit model by equivalencing the induced eddy current in the test object to a current flowing through an imaginary coil. In 2000, La et al. [41] discretized the eddy current distribution into current loops. By calculating impedance changes through a multi-transformer equivalent circuit, they achieved circuit model characterization for 3D defects. Subsequently, Lefebvre et al. [42] modeled PECT signals for non-ferromagnetic materials using an ideal transformer equivalent circuit model with nonlinear characteristics and employed the Levenberg–Marquardt algorithm for curve fitting. Their study highlighted that selecting appropriate initial guesses for model parameters is critical for fitting performance. However, the model directly equated the test object to an inductance coil and the induced eddy current to a single loop. This oversimplification compromised accuracy and universality, resulting in poor fitting performance for ferromagnetic materials.

The aforementioned circuit models and other concurrent improvements lacked in-depth consideration of coil interactions and the coupling relationship between the coil and the induced eddy currents. This led to significant modeling errors and limited generalizability. The Desjardins team [43,44,45,46,47] addressed these factors, constructing a PECT circuit model adapted for ferromagnetic materials. Their model demonstrated high computational accuracy and applicability, even for ferromagnetic materials and complex sensor structures. Xu et al. [48] estimated PECT equivalent circuit model parameters using system identification methods. They observed that the resistance and self-inductance of the secondary winding decrease monotonically with defect size, with self-inductance exhibiting higher sensitivity. This finding provided a new circuit parameter feature for quantitative defect evaluation.

Electromagnetic field models can be classified into analytical models and numerical models based on their solution methods.

### 4.2. Numerical Models

Among numerical calculation methods, the Finite Element Method (FEM) is the most widely adopted. Mature commercial software packages, such as Ansys and Comsol, can handle various PECT problems with appropriate configuration and tuning. Other computational methods include the Finite Difference Time Domain (FDTD) method and the Boundary Element Method (BEM). Recent research has primarily utilized FEM to investigate specific issues within PECT systems, focusing on magnetic field distribution, parameter optimization, feature evaluation, and sensor design. Yu et al. [49] employed FEM to study the variation laws of the eddy current field distribution during the simultaneous PECT inspection of tubing and casing. Shen et al. [50] validated pulsed remote field eddy current testing using a composite probe; by optimizing key parameters, they enhanced the ability to distinguish between defects on the inner and outer walls of pipelines. Zhang [51] investigated the influence of partial PECT model parameters on detection performance. Nafiah et al. [52] focused on the quantitative evaluation of inclined cracks, achieving simultaneous prediction of crack depth and angle. Additionally, Slobodnik et al. [53] examined the detection capability of differentially designed sensors regarding defect depth.

### 4.3. Analytical Models

Analytical models are solved through rigorous mathematical derivations, offering high calculation speeds and precision. Since PECT employs square wave or step excitation, time-frequency conversion and decomposition are typically performed using the Fourier transform or Laplace transform. Multiple harmonics are solved individually and then superimposed to obtain the final result.

In 1968, Dodd and Deeds [54] established analytical impedance models for eddy current testing coils, covering scenarios with coils above a two-layer conductive plate and encircling a two-layer conductive cylinder. Solved using the method of separation of variables, these models laid the foundation for subsequent PECT analytical solutions. However, the Dodd-Deeds models are overly cumbersome for complex objects, making accuracy and convenience difficult to control. Cheng [55] introduced the transfer matrix method, which greatly simplified the coefficient calculation process in the Dodd-Deeds model, thereby improving computational efficiency. Theodoulidis et al. [56] replaced the integral expressions in the Dodd-Deeds model with series expansions, significantly shortening calculation time and facilitating easier control of convergence.

As research has progressed, studies on PECT analytical models have deepened across various domains. Addressing defect types, the Theodoulidis team [57,58,59,60] constructed Green’s functions using the Truncated Region Eigenfunction Expansion (TREE) method and employed the surface integral method to model signals from surface cracks. Addressing the nonlinearity of ferromagnetic materials, they linearized the problem using the fixed-point iteration method and processed it via a modal approach, achieving outstanding performance in one-dimensional model calculations.

Regarding the effects of relative position changes, She et al. [61] based their work on the Dodd-Deeds model and employed the equivalent radius method to model the eccentricity between asymmetric cylinders and sensors, effectively reducing computational complexity. To address signal recovery, Xue et al. [62] utilized a frequency-domain model inversion algorithm based on Filon spline interpolation, optimizing the time resolution of time-domain signals obtained via the inverse Fourier transform.

Regarding the size of the test object, the flat plate approximation is no longer applicable when the diameter of a cylindrical specimen is less than five times the outer diameter of the sensor. For encircling sensors coaxial with the test object, models can be solved using the second type of Dodd-Deeds model [63,64]. For probe-type sensors placed parallel to the object surface with their axes perpendicular to the object, Mao et al. [65] applied second-order vector potential theory, while Chen et al. [66] established an equivalent analytical model to investigate solutions for single-layer pipelines. Extending the scope to double-layer cylindrical pipelines, Klein et al. [67,68] utilized MATLAB to derive a semi-analytical model for coils with axes perpendicular to the inner pipe surface.

## 5. Detection Signal Processing

**Figure 8:**
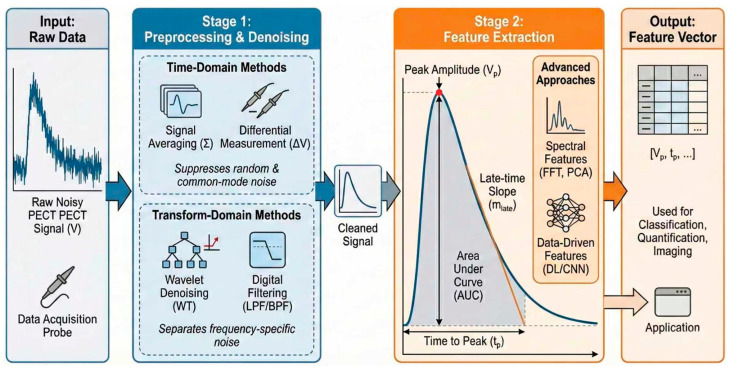
Schematic diagram of PECT signal processing method.

PECT signals contain information regarding the conductivity, magnetic permeability, and sensor lift-off distance of the object under test. However, the coupling of these factors, combined with noise interference, makes it difficult to directly extract target information from the detection signal. Consequently, signal processing is a core component of PECT. To systematically address these challenges, a standard PECT signal processing workflow typically encompasses two main stages, as illustrated in Figure 8. Stage 1 focuses on preprocessing and denoising the raw acquired signals. In this stage, time-domain methods (e.g., signal averaging and differential measurements) are employed to suppress random and common-mode noise, while transform-domain methods (e.g., wavelet denoising and digital filtering) are utilized to isolate frequency-specific interferences, ultimately yielding a cleaned signal. Subsequently, Stage 2 involves feature extraction, which is crucial for decoupling the desired parameters. Traditional approaches extract intuitive characteristics directly from the transient response curve, such as peak amplitude (V_p_), time to peak (t_p_), area under the curve (AUC), and late-time slope. Meanwhile, advanced approaches leverage spectral features (e.g., FFT, PCA) or data-driven deep learning models (e.g., CNNs) to mine high-dimensional information. Finally, these extracted parameters are assembled into a feature vector, serving as the essential input for downstream applications such as defect classification, quantification, and imaging.

### 5.1. Signal Preprocessing

In practical inspection, signals often exhibit offsets caused by non-defect factors, leading to errors in effective defect signals and affecting subsequent quantitative feature analysis. Common causes of such offsets include temperature variations, parameter fluctuations in circuit components, minor equipment vibrations, and environmental electromagnetic interference. Dang et al. [69] employed an array-based Ensemble Empirical Mode Decomposition (EEMD) method, which effectively eliminated the effects of background magnetic noise and temperature drift.

Noise can be categorized into random and non-random noise. Random noise, including electronic and inherent system noise, requires processing that preserves effective signal components related to defects. Common processing methods involve using differential sensors to suppress common-mode noise and applying various filtering techniques. In engineering applications, differential noise processing is classified based on the reference object: one uses the signal obtained in air, while the other uses the signal from a defect-free condition. Comparing the two, the latter not only suppresses common-mode noise but also effectively amplifies the proportion of defect information in the signal. Fan et al. [70] used a rectangular differential sensor to detect three types of damage in carbon fiber reinforced polymer (CFRP) plates; this approach corrected baseline drift and eliminated the need for reference signal measurement during new damage detection. Dung et al. [71] utilized differential Hall sensors to reduce interference from the primary magnetic field. This system performed well across multiple lift-off heights, significantly improving the signal-to-noise ratio (SNR) compared to single Hall sensors.

Beyond hardware optimization, random noise suppression includes time-domain and frequency-domain methods. Time-domain suppression primarily employs median filtering, though methods based on wavelet transform have also been proposed [72]. Based on multi-coil coupling theory, Huang et al. [73] proposed a PECT signal model for ferromagnetic materials. By transforming the signal into a double-logarithmic domain and applying median filtering, they effectively improved the SNR. Subsequently, addressing issues with model fitting performance, Huang et al. [74] adopted numerical cumulative integration for noise processing. This approach avoided signal distortion caused by filtering and enhanced noise processing effectiveness. In frequency-domain processing, signals are typically sampled and converted to digital form, then transformed via Discrete Fourier Transform (DFT) to separate noise based on frequency differences. However, since PECT signals lack strict periodicity, spectral leakage inevitably occurs during sampling, causing energy to spread across adjacent frequency points [75,76]. Huang et al. [77] effectively suppressed DFT spectral leakage by enforcing strict periodic matching between sampling points and signal frequency.

### 5.2. Feature Extraction

PECT signals contain multidimensional information about the object under test; extracting this information from the signal waveform is a critical step in signal processing. A common approach involves selecting appropriate features in the time-frequency domain, which directly or indirectly reflect defect characteristics. Based on the time-domain waveform and the magnetic properties of the object, features are categorized into two groups. For ferromagnetic materials, typical features include the −3 dB point, knee point, and late-stage signal slope. For non-ferromagnetic materials, features include the peak point, peak time, zero-crossing time, and lift-off intersection (LOI) point.

In addition to statistical methods [78], machine learning algorithms [79]—particularly various architectures of artificial neural networks (ANN) [80] such as Convolutional Neural Networks (CNN) [81], Backpropagation (BP) networks [82], and Spiking Neural Networks (SNN) [83]—are extensively employed in signal processing. These algorithms are typically combined with feature extraction to enhance precision, though some studies utilize them for end-to-end signal processing. Yan et al. [84] proposed the “average peak value” as a novel feature for pulse-modulated eddy current signals and generated images based on this feature. By combining an improved small sub-domain filtering method with the Canny algorithm for corrosion profile recognition, they achieved an innovative integration of feature extraction, filtering, and image processing, resulting in high recognition accuracy and noise immunity. For defects in laser-welded structures, He et al. [85] introduced features such as the fundamental amplitude, the ratio of peak to rising edge curvature, the amplitude ratio of fundamental to third harmonics, and the marginal spectrum peak. By quantitatively correlating these features with defect type and size, they established a defect recognition model with an accuracy of approximately 90%. She et al. [86] selected the high-frequency components of the signal after Discrete Fourier Transform (DFT) as features and combined them with deep learning. This approach achieved classification accuracies exceeding 95% for defect size, sample thickness, and defect depth. Addressing the issue of noise interference in traditional features for deep defects, Rao et al. [87] proposed two new features—the ratio of the detection signal to the reference signal and the time constant—for defect detection and separation. In addition to the aforementioned methods, other studies utilize techniques such as the combination of DFT and Wavelet Transform [88], Wavelet Transform alone [89], Short-Time Fourier Transform (STFT) [83], and Hilbert-Huang Transform (HHT) [90] for preliminary processing prior to feature extraction.

**Figure 9:**
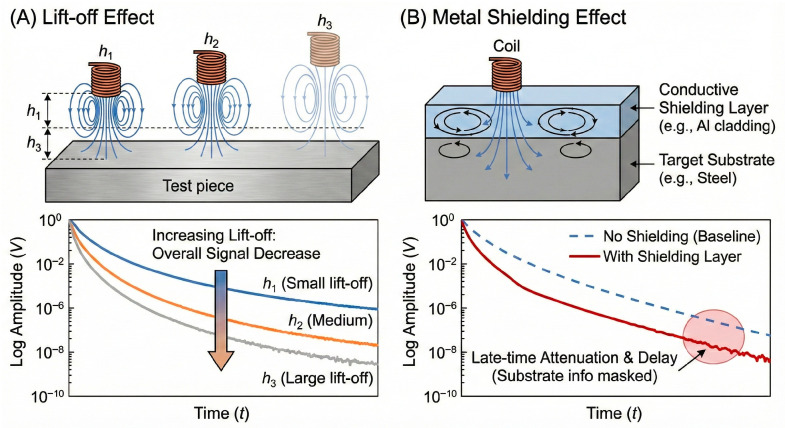
Influence of lift-off effect and metal shielding effect.

In practical PECT applications, both the lift-off effect and the metal shielding effect severely degrade signal quality, albeit through distinct physical mechanisms, as comparatively illustrated in Figure 9.

The lift-off effect, caused by the thickness of the test object itself or by various protective and coating layers, significantly reduces PECT accuracy and degrades depth detection capability. As explicitly shown in Figure 9A, an increase in lift-off distance (e.g., from h_1_ to h_3_) primarily results in a global decrease in the overall signal amplitude across the transient response due to the spatial attenuation of the magnetic field. Despite this amplitude drop, specific signal features can remain robust. For non-ferromagnetic materials, Giguère et al. [91] discovered that the Lift-Off Intersection (LOI) point in the signal waveform is independent of lift-off height. For ferromagnetic materials, the linear decay rate, obtained by linearly fitting the late-stage signal slope in a semi-logarithmic coordinate system, remains unaffected by the lift-off effect. However, not all detection signals can be processed using these two methods. Since defect information is distributed across other features, alternative methods are required to suppress the lift-off effect. Common suppression techniques generally rely on feature extraction and processing to attenuate the influence of lift-off. Fu et al. [92] plotted dynamic signal trajectories in the complex plane using Fast Fourier Transform (FFT). By separating the defect component and translating the defect trajectories of various harmonics to the zero-lift-off point, they reconstructed the signal via Inverse Fast Fourier Transform (IFFT), thereby eliminating the lift-off effect. Duan et al. [93] employed a linear combination of features from different lift-off heights, achieving significant suppression effectiveness within a 1.2 mm range. Addressing the large and non-uniform lift-off caused by non-ferromagnetic metals and their protective layers, Wang et al. [94] proposed a Dynamic Apparent Time Constant (D-ATC) method based on a coil coupling model. By analyzing the time and amplitude characteristics of the D-ATC curve, they correlated the dynamic eddy current changes with the geometric dimensions of the non-ferromagnetic metal, effectively suppressing the lift-off effect.

Variations in lift-off height introduce additional challenges. In PECT, exceeding a specific lift-off variation threshold causes the sensor’s induced response to become dependent on the specimen’s conductivity. Jin et al. [95] demonstrated that this threshold can be flexibly controlled by adjusting the horizontal and vertical distances between the excitation and receiving coils. They proved a linear relationship between these two parameters and the threshold, resolving the lift-off limitation issue during the inspection of different specimens.

The metal shielding effect, primarily caused by metallic coatings or wire meshes, not only exacerbates the lift-off effect but also diverts excitation magnetic field energy away from the target object, leading to reduced detection accuracy. As depicted in Figure 9B, unlike the simple amplitude drop caused by lift-off, a conductive shielding layer generates its own prominent eddy currents. This masks the secondary field from the underlying target substrate, leading to severe late-time signal attenuation and delay, which drastically reduces detection accuracy. Xu et al. [96,97] found that aluminum or stainless steel wire meshes within insulation layers have a negligible impact on eddy currents in steel plates. However, low-carbon steel meshes expand the effective detection area of the probe while reducing eddy current density, signal amplitude, and decay rate, thereby affecting detection precision. To mitigate the metal shielding effect, some studies focus on sensor structure optimization. Zhang et al. [98] analyzed the characteristics of Transmit-Receive (TR) sensors using spatial spectrum and analytical models, proving that TR sensors can effectively weaken the shielding effect caused by zinc coatings. Other approaches involve using permanent magnets to magnetize the coating. Demers-Carpentier et al. [99] introduced a novel PECT probe designed for inspecting through ferromagnetic metal coatings. Permanent magnets were used to saturate the metal coating, reducing its relative permeability in the coverage area and thereby diminishing the shielding effect. Additionally, signal processing methods, particularly Principal Component Analysis (PCA), have been adopted to reduce shielding influence. Nafiah et al. [100] used PCA to separate the effects of the coating from signal features, enhancing detection accuracy. Conversely, the metal shielding effect can be utilized positively in sensor design. Mirzaei et al. [101] designed a novel eddy current speed sensor with ferromagnetic shielding. The added shielding layer improved sensor sensitivity and reduced the impact of interference from nearby ferromagnetic objects. Figure 9 illustrates the effects of lift-off and metal shielding on the amplitude and stability of the detected signal.

Despite the significant achievements in PECT signal processing, existing methodologies still exhibit notable limitations that hinder their broader industrial application. First, while traditional signal processing methods possess high interpretability, they often fail under complex multi-parameter coupling conditions. For instance, when the lift-off effect and the metal shielding effect vary simultaneously, extracting effective features using traditional statistical methods becomes exceedingly difficult.

Second, although machine learning algorithms—particularly deep neural networks such as Convolutional Neural Networks (CNN) and Spiking Neural Networks (SNN)—have demonstrated remarkable classification accuracies (often exceeding 95%), they inherently suffer from the ‘black-box’ problem. These data-driven models lack physical interpretability, making it difficult to trace the physical origins of specific defect features. Relying heavily on empirical data mapping rather than theoretical physical mechanisms poses significant challenges for further targeted improvements. Furthermore, training robust deep learning models requires massive amounts of high-quality, labeled empirical data. This is not only highly expensive but also sometimes virtually impossible to acquire for rare defect morphologies in complex structures, such as insulated cryogenic pipelines.

## 6. Applications and Development of PECT

Thanks to its superior versatility and detection precision, PECT is widely applied in the fields of material characterization and defect detection. Furthermore, hybrid applications of PECT with other detection technologies, along with continuous improvements in detection methods, are being actively proposed. The following sections will elaborate on these aspects.

### 6.1. Material Characterization and Defect Detection

In the realm of material characterization, PECT is primarily utilized to measure the electrical conductivity and magnetic permeability of materials. In defect detection, the process typically begins with thickness measurement to achieve basic defect identification, followed by an evaluation of defect size. Thickness measurement plays a pivotal role in this process, encompassing both the thickness of the object under test and that of various protective layers.

Wang et al. [102] analyzed the interactions among coating conductivity, coating thickness, and PECT signals. They proposed methods for measuring the thickness and conductivity of single-layer conductive coatings, as well as a method for detecting the thickness of double-layer conductive coatings. Lin et al. [103] investigated PECT signals in non-ferrous metal plates using a hybrid input signal containing both short and long pulses. They discovered that conductivity could be estimated from the peak value of the short-pulse response; once the conductivity was known, the plate thickness could be derived from the rise time of the long-pulse response. Chen et al. [104] measured the conductivity and relative permeability of ferromagnetic materials by establishing a least-squares relationship between the measured and theoretical values of the time-domain induced voltage in the pulsed eddy current field. They also explored the distribution laws of electromagnetic parameters in common steels. Huang et al. [105] proposed a method combining swept-frequency eddy current measurement with machine learning techniques to simultaneously estimate the magnetic permeability, conductivity, thickness, and lift-off of metal plates. This approach further improved detection accuracy, keeping the relative error within 3.5%.

### 6.2. Hybrid Applications and Detection Improvements

With the further expansion of the global industrial scale, novel composite materials are being extensively applied in fields such as aerospace, energy, and biomedicine, imposing new requirements on detection technologies. Against this backdrop, hybrid applications of PECT with other technologies, as well as improvements based on PECT, have seen further development.

In terms of hybrid technical applications, Wu et al. [106] employed eddy current thermography, combining electromagnetic induction with infrared thermal imaging to achieve visual defect detection. This study also utilized DC-biased magnetization to enhance permeability distortion within the skin layer, thereby improving the thermal contrast between defective and defect-free regions and further boosting detection capability. Zheng et al. [107] combined PECT with Electromagnetic Acoustic Transducer (EMAT) testing, designing a novel hybrid probe for marine equipment inspection. This approach effectively enhanced the capability to detect near-surface defects in materials.

Regarding PECT technological improvements, Xie et al. [108] proposed a frequency-band-selecting PECT method. By selecting excitation within a specific frequency range to detect wall thickness loss at specific depths, this method offers higher detection sensitivity and controllability compared to traditional PECT methods.

Despite the promising prospects demonstrated by these hybrid applications, current multimodal detection systems still exhibit significant limitations that hinder their widespread industrial adoption. In most existing approaches (such as the combination of PECT with thermography or EMAT), different modalities primarily operate in parallel rather than being deeply integrated. Hardware designs often merely co-locate distinct sensors or simply package them into a single component without synergistic physical design. At the algorithmic level, current systems typically process signals from each modality independently, lacking cross-domain data fusion algorithms capable of simultaneously processing multiphysics data (e.g., tightly coupling electromagnetic and acoustic responses to decouple complex defect morphologies). Furthermore, not all multimodal combinations yield positive synergistic effects. For instance, when investigating the joint application of permanent and alternating magnetic fields for inspecting thick-walled steel products, Reutov [109] found no distinct advantages in supplementing traditional Magnetic Flux Leakage (MFL) methods with eddy current sensors, additionally citing unacceptably high energy consumption. This further demonstrates that future hybrid techniques must pursue profound physical coupling rather than mere hardware superimposition. Transitioning from simple ‘sequential multi-sensor inspections’ to genuine ‘deep data fusion’—where multiple physical fields collaborate to overcome the inherent bottlenecks of individual techniques—remains a formidable challenge.

## 7. Conclusions

This paper has reviewed PECT technology from the perspectives of principles, detection sensors, modeling methods, signal processing, and technical applications and development. It is evident that PECT technology is closely integrated with practical engineering applications, with its development direction directly driven by engineering needs. To further evolve amidst the inspection demands of the new energy era, PECT technology must transcend traditional research paradigms, leveraging its inherent advantages while actively embracing new technologies. It is recommended that future research and improvements focus on the following areas:

(1) Research on PECT Mechanisms. Previously, PECT focused primarily on ferromagnetic metal materials. However, with the development of new energy sources—particularly the large-scale application of LNG, methanol, and liquid hydrogen—the demand for inspecting non-ferromagnetic metals such as stainless steel is rising continuously. Research into PECT mechanisms should consider the characteristics of non-ferromagnetic metals, as well as the effects of insulation layers, metallic anti-corrosion cladding, and temperature variations caused by medium flow. Furthermore, PECT research could extend to the inspection of buried metal pipelines. Introducing heterogeneous soil into the multiple electromagnetic wave transmission interfaces to study the detection mechanism for shallow-buried metal pipelines represents a key node in the current development of nondestructive testing.
(2) Further Improvement of Detection Sensors. Existing sensors are mostly limited to single-structure optimization, exhibiting insufficient adaptability to irregularly shaped components and complex large-scale equipment, as well as needing improvement in anti-interference capabilities. Current research could leverage novel materials such as nanocrystalline soft magnetic alloys and flexible conductive polymers to construct smart sensors with multi-parameter perception. This would enable real-time sensor self-calibration and adaptive parameter adjustment based on the electromagnetic characteristics of the test object. Alternatively, improvements could focus on anti-interference capabilities by integrating high-performance magnetic shielding layers to suppress environmental electromagnetic noise.
(3) Fusion of Multiple Detection Technologies. PECT focuses primarily on metal component inspection and has poor adaptability to composite materials, yet such new materials often come with extremely high inspection requirements. It is worth considering promoting the fused development of PECT with other detection technologies—such as visual innovations in pulsed eddy current thermography—to absorb the strengths of other techniques and enhance detection capabilities. Additionally, further deepening the application of deep learning and image processing technologies in PECT is advisable. This involves improving detection precision and intelligence levels and proposing new data-driven signal processing methods to eliminate the influence of lift-off and cladding.

## Data Availability

No new data were created or analyzed in this study. Data sharing is not applicable to this article.
